# Of mice and men: opposing effects of nicotinamide riboside on skeletal muscle physiology at rest and during exercise

**DOI:** 10.1113/JP281428

**Published:** 2021-03-08

**Authors:** Laís S. S. Ferreira, Evandro A. De‐Souza

**Affiliations:** ^1^ Institute of Neuropathology University Medical Centre Freiburg Freiburg Germany; ^2^ Faculty of Biology University of Freiburg Freiburg Germany; ^3^ Neurobiology Division MRC Laboratory of Molecular Biology Cambridge UK

**Keywords:** exercise, human physiology, metabolism, nicotinamide riboside, skeletal muscle

## Background

Physical exercise is one of the most efficient and researched strategies available for improving human health span. As a way of sustaining muscle contraction during exercise, cellular mechanisms drive resources to support ATP production. The balance between the oxidized and reduced forms of NAD is important for sustaining energy‐producing metabolic reactions. NAD^+^ is a coenzyme involved in pleiotropic cellular processes, including the regulation of various metabolic enzymes but also chromatin remodelling and DNA repair. In rodents, NAD^+^ levels have been shown to decrease with ageing due to a rise in the activity of the CD38 NAD glycohydrolase enzyme driving age‐related phenotypes, such as the decay of mitochondrial function (Yoshino *et al*. 2018). Thus, because of its possible therapeutic applications, NAD^+^ dietary supplement precursors such as nicotinamide riboside (NR) gained a lot of attention (Yoshino *et al*. 2018). Although the findings are promising, they are primarily based on studies performed in invertebrates and mammalian models, while a subset of recent studies indicates that the effects of NAD supplementation in humans might be much more subtle than what was observed in the preclinical studies. For example, dietary NR supplementation failed to alter mitochondrial physiology in the skeletal muscle of obese individuals (Dollerup *et al*. 2020), conflicting with the positive findings in mice.

## Nicotinamide riboside supplementation has no effect on the mitochondrial physiology of human muscle

In a recent study published in *The Journal of Physiology*, Stocks and colleagues investigated the effects of 1 g NR per day on skeletal muscle physiology in eight young male individuals at rest or during exercise (Stocks *et al*. 2021). The authors’ hypothesis was that prior to acute exercise, NR treatment would increase NAD pools and, subsequently, boost post‐exercise signalling responses. Based on previous research on the effects of NR on the availability of human skeletal muscle NAD^+^, a 1‐week supplementation with NR was chosen. After that, participants were subjected to exercise to exhaustion on a bicycle ergometer. The participants had their physical endurance evaluated and skeletal muscle biopsies were collected to determine the consequences of NR supplementation on post‐exercise signalling responses, including several metabolic and mitochondrial parameters. The authors found no changes between groups in cardio‐respiratory performance parameters after 1 week of supplementation with NR compared to the group that received a cellulose placebo. In addition, the levels of different metabolites commonly modified during physical exercise, such as plasma non‐esterified fatty acid, glucose, glycerol and lactate, were unaffected at rest or with exercise.

Alterations in mitochondrial physiology and biogenesis are amongst the remarkable effects of NR supplementation in preclinical studies. Therefore, the authors investigated if NR would be able to promote any mitochondrial physiological alterations with biopsies of the skeletal muscle. Neither exercise nor NR had significant effects on mitochondrial oxygen consumption nor on the levels of different mitochondrial proteins. The group also monitored whether NR treatment for 7 days could modify the levels of sirtuin (SIRT1 and SIRT3) in human skeletal muscle, as these are highly modulated by NAD^+^ dietary supplements in rodents, as well as mediators of the beneficial outcomes of these molecules in the rodent models. SIRT1 and SIRT3 activity were accessed by measuring the levels of acetylation of their targets (p53^Lys382^ and MnSOD^K122^, respectively) along with the global levels of protein acetylation, but none of these parameters were affected by NR during rest or exercise.

It is known that physical exercise induces a series of signalling events in the skeletal muscle, especially in nutrient sensing pathways. Individuals presented a significant increase in the phosphorylation levels of AMPK^Thr172^ and ACC^Ser79^ after exercise. The same degree of induction was observed with individuals treated with NR. There was also not observed any differential activation by NR on the mRNA levels of *PPARGC1A*, which encodes the transcriptional factor peroxisome proliferator‐activated receptor γ coactivator 1α, a master regulator of mitochondrial metabolism. In other words, different from what is observed in rodent studies, NR does not seem to mimic or boost the effects of physical exercise on the activation of different nutrient signalling pathways in skeletal muscle.

A possibility to explain the absence of effects of NR would be that the chosen dosage was not sufficient to promote significant alterations in the metabolism of NAD^+^ in skeletal muscle. To test this possibility, the skeletal muscle NAD‐metabolome was evaluated by liquid chromatography–mass spectrometry. Surprisingly, there were no significant differences in the levels of NR or NAD^+^ between the groups that might explain why the authors were unable to observe differences in the activity of SIRT1 and SIRT3. One possibility is that the regulation of NAD^+^ levels and NR recycling is more severely controlled in humans and that other concentrations or strategies will be required to modify these metabolite levels. Nonetheless, they were able to detect a rise in nicotinic acid mononucleotide, Nar, Me2PY and Me4Py levels, strongly indicating that 1 week of NR treatment was sufficient to alter the human skeletal NAD metabolome. In future studies, it will be interesting to include the measurement of NADH levels. Although the total levels of NAD^+^ are unchanged, one possibility is that the NAD^+^/NADH balance could be influenced by NR treatment. Finally, the authors measured the transcriptional level of some genes of the NAD^+^ synthesis and salvage pathway. They were able to identify that physical exercise increases the expression of the gene for nicotinamide *N*‐methyltransferase (NNMT) – a methyltransferase of nicotinamide – and that this activation is blunted by treatment with NR. Further studies with NR in humans should validate this finding in other cohorts and investigate the physiological relevance of the differential modulation of the expression of *NNMT* by NR. Interestingly, in rats, the regulation of NNMT activity during exercise has already been shown to influence the performance of animals during anaerobic exercise (Zhou *et al*. 2018). Finally, another point to be considered is that *S*‐adenosyl methionine is a substrate for the NNMT reaction and it could also be modulated within NR treatment.

## Final considerations and future questions

In concordance with Stocks *et al*. (2021), a contemporary study using the same dosage of NR for 21 days in aged individuals at rest also did not reveal alterations in mitochondrial physiology (Elhassan *et al*. 2019). Conversely, the NAD^+^ metabolome was also found to be augmented in elderly by NR. However, RNA sequencing of human skeletal muscle detected downregulation in genes of glycolytic, tricarboxylic acid and even mitochondrial pathways after NR supplementation, but surprisingly, no key changes in NAD^+^ metabolism genes were found. It is possible that subtle transcriptional changes could be found in the study of Stocks *et al*. (2021) by employing high throughput techniques. Based on these observations, it will be interesting to understand the mechanisms of how nicotinamide riboside can modulate the gene expression signature of human skeletal muscle cells, at least for aged tissues, if it is not affecting NAD^+^ or mitochondrial physiology. In future studies, one key point to be considered is whether NR treatment would benefit young healthy individuals, since they have normal levels of NAD^+^. Knowing exercise partially restores the health of aged tissues and that NR might have positive responses in elderly, a promising next approach for a clinical trial would be to evaluate the combined effects of exercise and NR supplementation during ageing (Custodero *et al*. 2020).

Finally, this study raises a series of questions for the field. For instance, why is NR supplementation not so successful in altering mitochondrial function in human skeletal muscle when compared to rodent studies? Studies with larger and more diverse cohorts and with longer periods of treatment are required to enlighten this issue. A promising venue for future research would be to explore the impact of NR on other aspects of physical activity, such as fat mobilization and mitochondrial function in other tissues. It is also possible that species‐specific optimal strategies to increase NAD^+^ levels with external sources exist. Future studies could investigate the role of other NAD^+^ dietary sources, such as dihydronicotinamide riboside, which is successful in boosting NAD^+^ on rodents through the action of adenosine kinase.

## Conclusion

Taken together, the study of Stocks and colleagues showed that 1 week of NR supplementation has no identifiable effects on human skeletal muscle during rest or exercise. Nevertheless, further studies using other demographic populations and treatment for longer periods of time are necessary to establish the real potential of NR for modifying human physiology.

## Additional information

### Competing interests

No competing interests declared.

### Author contributions

Both authors have read and approved the final version of this manuscript and agree to be accountable for all aspects of the work in ensuring that questions related to the accuracy or integrity of any part of the work are appropriately investigated and resolved. All persons designated as authors qualify for authorship, and all those who qualify for authorship are listed.

### Funding

L.S.S.F. has received funding from the European Union's Horizon 2020 research and innovation programme under the Marie Sklodowska‐Curie grant agreement no. 813294. E.A.deS. has received funding from the European Union's Horizon 2020 research and innovation programme under the Marie Skłodowska‐Curie grant agreement number 894039.
